# How the wide awake approach is changing hand surgery and therapy

**DOI:** 10.1186/1753-6561-9-S3-A80

**Published:** 2015-05-19

**Authors:** Donald Lalonde

**Affiliations:** 1 Department of Plastic and Reconstructive Surgery, Dalhousie University, Saint John, New Brunswick, Canada, E2K 1J5

## 

The wide-awake approach to flexor tendon repair has decreased our rupture and tenolysis rates, and permitted us to get consistently good results in cooperative patients. We no longer perform flexor tendon repair with the tourniquet, sedation and muscle paralysis of general or block (Bier or axillary) anesthesia. Injection of only lidocaine with epinephrine wherever incisions will be made in the finger and hand permits comfortable tourniquet-free awake patients cooperate with active finger full flexion and extension testing during the surgery.

5 main reasons that I never want to do a flexor tendon repair asleep or with a motor block ever again

## 1) Avoid gap and rupture

When a patient takes the tendon repair through a full active range of motion during the surgery, 7% of the time, the surgeon will observe a gap that occurs because the tendon suture is not tight enough. The gap can be repaired with a tighter suture before the skin is closed to avoid rupture. In our series of 102 patients with intraoperative testing of wide awake flexor tendon repair, 7 patients showed intraoperative gapping that was repaired before the skin was closed. They did not rupture postoperatively. None of the 102 patients who followed proper postoperative instructions with true active early protected movement ruptured after surgery. (*Higgins A*, *Lalonde DH*, *Bell M*, *et al. Avoiding flexor tendon repair rupture with intraoperative total active movement examination. Plast Reconstr Surg 2010; 126:941–5.*)

## 2) Seeing a full fist of flexion and full active extension during the surgery without gap gives us the confidence to know they will not rupture with half a fist of flexion and half full extension with protected movement 3 days after the surgery

We no longer use Kleinert rubber bands or “place and hold” with protected post-operative active movement. True active movement, just enough to keep the tendon gliding a little, is giving us better results.

In the first 2-4 days after surgery, we keep the hand elevated and immobile in a splint with the wrist in comfortable 25-45º of extension (not flexed or neutral). The MP joint is flexed to a comfortable 45ºto 80º from full extension, and the PIP and DIP joints are fully extended. Collagen formation does not start until day 3, so serious adhesions will not start before then. We avoid immediate movement after surgery as this generates bleeding inside the wound which adds to the scar tissue. Also, the finger and tendons are swollen and have more friction as they try to move in the sheath in the first 3 days. Elevation and immobilization decrease bleeding and swelling in the first days after surgery.

Day 3 to 4 weeks after surgery, they stay in the splint but come out hourly for early protected movement hourly while awake if the fingers are not swollen and hurting, and if they are off all pain medication. They passively warm up the MP, PIP, and DIP joints with flexion to decrease friction and work of the tendons before starting active movement. We allow up to half a fist of midrange active flexion of the fingers and focus on full active extension of the PIP and DIP joints. (45-90ºof active flexion of MP, 0-45ºof active flexion of the PIP and DIP joints). We do not allow full active or passive full flexion of the PIP and DIP joints (45-90º) because this would increase the friction across the repair as well as increase the risk that the tendon repair site flex at 90ºor more and get caught on the proximal hard edge of a fully flexed A4 pulley and pulling the repair apart. We have seen this problem with active movement during surgery and solved it by dividing the A4 pulley. We tell patients: “You can move it but you can’t use it and you can’t do what hurts.”

**Figure 1:**
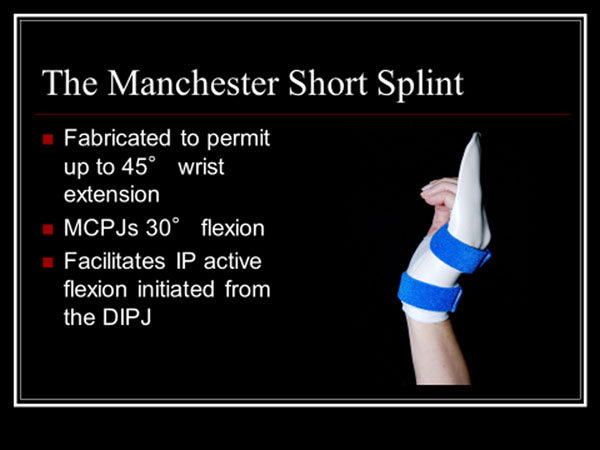
Photo compliments of Fiona Peck, Manchester, England

## 3) Decrease the need for tenolysis

We almost never need to do tenolysis after flexor tendon repair now. The reason is that we make sure we have a full range of active flexion and extension before the skin is closed so that tenolysis is less likely to be needed postoperatively. If the repair does not fit through the pulleys during the surgery, it is not likely to fit through the pulleys later and will almost certainly require a tenolysis.

After the repair, the awake comfortable patient with no tourniquet is asked to flex and extend the finger completely. If the repair gets caught on pulleys, we incrementally vent (divide) only as much pulley as we need to in order to see full active flexion and extension. We agree with Jin Bo Tang that the entire A4 pulley and up to half of the distal A2 pulley can be divided without causing bowstringing if most of the cruciate pulleys are preserved. What you see with active movement during surgery is what you get. It changes the whole operation.

An alternative to venting pulleys is to trim the repair or slim it with more sutures so it can fit through the pulleys before you close the skin.

## 4) You get at least a full hour during which the surgeon (and the therapist) can educate the patient during the surgery

An educated patient is much more likely to do what he should right after surgery than one who wakes up with a freshly repaired flexor tendon and has no idea what he should or should not be doing. In addition, the awake patient has seen his finger move well during the surgery and knows it will work if he follows the instructions given to him during the surgery. Seeing is believing.

I talk to my patients during the surgery and explain to them how to look after their hand after surgery. We also have our therapists come into the clinic room where we do our surgery to intraoperatively assess the patients, see the repaired structures moving, and educate the patient before and during skin closure. The patient gets to meet and know the therapist during the surgery.

## 5) You get to see the effect of repairing superficialis in zone II and deciding whether or not to keep that repair

We first repair profundus and be sure we see full active movement with no gapping. If the repair of superficialis seems like it will be possible or helpful, we go ahead and do it and retest full active fist flexion and extension. If the movement is downgraded by repairing superficialis, we will take down that repair. There is currently debate about to repair or not repair superficialis. Seeing the movement during the surgery takes away all debate because seeing movement is understanding results.

